# Core Processes: How to Use Evidence, Theories, and Research in Planning Behavior Change Interventions

**DOI:** 10.3389/fpubh.2020.00247

**Published:** 2020-06-24

**Authors:** Robert A. C. Ruiter, Rik Crutzen

**Affiliations:** ^1^Department of Work and Social Psychology, Maastricht University, Maastricht, Netherlands; ^2^Department of Health Promotion, Maastricht University, Maastricht, Netherlands

**Keywords:** Core Processes, applying theories, applied psychology, behavior change, problem-driven approach

## Abstract

Psychology is not only a basic behavioral science but also an applied discipline that is used to solve societal problems. In a problem-driven context, the search for existing literature, the correct application of appropriate theories, and the collection of additional research data are basic tools essential for the systematic development of any theory- and evidence-based behavior change intervention. The processes of brainstorming, literature review, theory selection and application, and data collection are “Core Processes” that can be used in different phases/steps of intervention planning—from needs assessment to intervention design to program implementation and evaluation—and within different planning frameworks. In this paper, we illustrate how the use of these “Core Processes” provides expert, empirical and theoretical guidance to planners from problem definition to problem solution. Specific emphasis is put on finding theories that are potentially useful in providing answers to planning questions using a combination of approaches to access and select theories (i.e., the topic, concept, and general theories approaches). Furthermore, emphasis is put on the logic of answering planning questions in a specific order by first brainstorming before consulting the literature, then applying theories, and finally collecting additional data.

Within social and health psychology teaching programs at institutes of higher education, we train students to become experts in the understanding and promotion of behaviors that contribute to better population health, public safety, and sustainable environments. Graduates of such programs are seen as experts on behavior change. They are expected to make informed decisions when it comes to identifying targets for behavior change interventions, selecting appropriate change methods to reach these targets, and translating these methods into practical applications, while making sure these measures can be implemented and their effectiveness can be assessed. Expertise in intervention planning implies that planners not only know about information sources that could help them in finding answers to the above questions, but also are able to translate the information gained from these sources in such ways that the final answers are indeed informed by expert opinion, empirical research, and theory, thus increasing the likelihood of selecting relevant intervention goals and effective and feasible intervention content [cf. (1–3); for empirical evidence, see for example (4)].

In the Netherlands, and many other countries, most of the psychology programs include a practical training on applying psychological theory. In such skills training, students select theory- and evidence-based explanations for practically relevant problems in which behavior plays a prominent role, such as in the prevention of infectious diseases (e.g., HIV infection) and the promotion of healthy lifestyles (e.g., sufficient exercise), the early detection of life-threatening diseases (e.g., cancer, diabetes), promoting adherence to therapy and medical regimes to prevent disease episodes (e.g., asthma) or even death (e.g., AIDS), or problems in the domains of sustainability (e.g., energy conservation) or safety (e.g., fire prevention). These explanations are found through a systematic process of asking a question (e.g., why do people perform behavior X?), brainstorming possible answers, looking for empirical evidence and theoretical support, conducting new research, and coming to a final list of answers to the question. This working method is originally described by Veen (5) and in later years has been transformed into the PATH protocol (6, 7). However, this systematic process to finding answers to questions—here referred to as Core Processes—is not limited to the understanding of problematic behaviors, but extends to the full process of intervention planning from analyzing the problem and risk behavior at hand, to selecting methods of change, to designing implementable and evaluable interventions (8).

In intervention planning, there are different frameworks available [see O'Cathain et al. (2) for a taxonomy of planning frameworks], such as PRECEDE-PROCEED (9), the Behavior Change Wheel (10), and Intervention Mapping (11), that provide guidance to planners from problem definition to problem solution. Across all these planning frameworks, applied psychologists may encounter the difficulty of using expert knowledge, empirical evidence and theory in order to analyze the problem and inform behavior change interventions. Brainstroming, reviewing existing literature, applying appropriate theories, and collecting additional research data are basic tools (Core Processes) in different phases/steps of planning frameworks, but often it is unclear exactly how and when these processes should be used in problem analysis and solving (6–8).

Here, *Core Processes* are presented as a helpful and systematic way to answer questions during intervention design. We would like to stress that although these Core Processes are described within Intervention Mapping (11), they can be applied in any planning framework and in each step of program planning from problem analysis, to intervention design, to program implementation and evaluation. Therefore, Core Processes are not a planning framework on their own, but a helpful and systematic approach to answer questions relevant to problem definition and solution using expert knowledge, empirical evidence and theory, and collecting additional data. The use of Core Processes is essential within problem-driven applied psychology because too often intervention planners claim to have reviewed empirical literature, applied theories, and collected additional data, but in fact have done these tasks incompletely and selectively. For example, when not making explicit links between determinants and methods of change or making incorrect translations from change methods to their practical applications (12). Also, in our teaching and consulting activities planners indicate finding it difficult in practice to apply these Core Processes correctly and sufficiently.

## Theory-Driven and Problem-Driven Applied Psychology

Within applied (health and social) psychology, a distinction can be made between two approaches: theory-driven and problem-driven applied psychology (13). Theory-driven applied psychology involves testing a theory in an applied setting, primarily in order to gain insight into the external validity of the theory. Problem-driven applied psychology refers to scientific activities that focus on changing or reducing a practical problem. In problem-driven applied psychology, theories are used, but problem solving is the primary focus of this approach, and the criteria for success are formulated in terms of problem reduction, with contributions to theory as a useful by-product. Problem-driven applied psychology is an important field, because it provides an ultimate test for the usefulness of psychology both as a discipline and as a profession.

## Core Processes for Using Theory and Evidence

Processes involved in answering a question using empirical data and theory can be complex and time-consuming; sometimes planners do not persevere in working through these difficulties. Consequently, the understanding of a problem is often incomplete, and attempts to solve the problem may be based on faulty premises/assumptions. Also, the problems that are addressed are often complex and require a multidisciplinary approach. For example, the Focus on Strength project combined existing ideas, evidence and theory from biological and psychological perspectives and introduced strength exercises to counter the negative health consequences associated with obesity and ensure high participation motivation in overweight youth (14). Furthermore, behavior change is difficult by definition: If it was easy, we would not need experts in change. So, although the required expertise within multidisciplinary planning groups may vary based on the problem that is addressed, expertise in behavior change (e.g., an applied psychologist) is always required.

**Figure 1:**
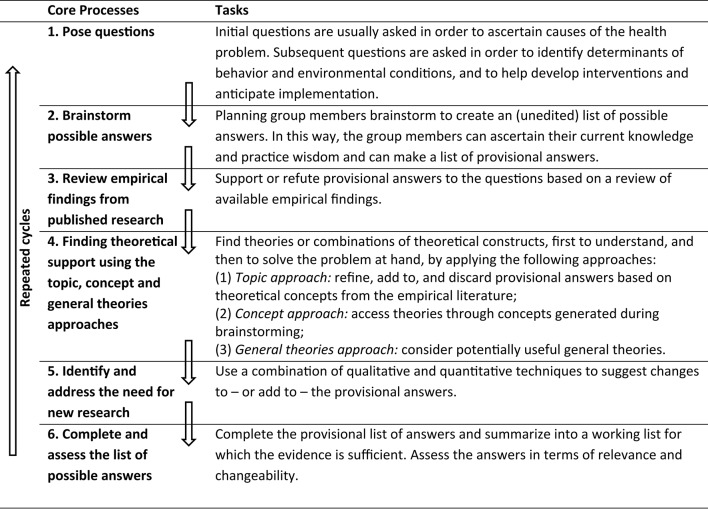
Core Processes for Using Evidence, Theories, and Research.

Using Core Processes minimizes the likelihood of incomplete understanding and selecting ineffective solutions. As depicted in Figure 1, Core Processes includes six steps that are described below. This is followed by an example of how to apply Core Processes in intervention planning. However, before describing Core Processes, it is important to stress that unlike planning frameworks for intervention development that generally are meant to be iterative and flexible in the order of steps (e.g., intervention planning may start from an already existing intervention implemented in a different intervention population and context), Core Processes has a fixed order of six steps, starting with asking a question, followed by first consulting with experts, then reviewing the existing empirical evidence and finding theoretical support, and if then still needed collect additional data. By keeping this order of steps, it is guaranteed that the knowledge that is available to answer planning questions is indeed accessed and new research is both relevant and informative.

### Step 1. Pose Questions

The first step when following the Core Processes is to pose (the right) questions. The first questions are often asked as a means of analyzing possible causes of the health problem (e.g., what are important risk behaviors?). Later questions are used to identify determinants of behavior and environmental conditions, and help to both develop interventions and plan intervention implementation. It is crucial that the planning group is on the same page regarding *which* question needs to be answered at *what* moment (e.g., problem analysis, identifying determinants, selecting change methods, designing implementation strategies), before continuing with the second step of the Core Processes. Lack of clarity about the questions that have to be answered might lead to a feeling of being lost in translation during subsequent steps.

### Step 2. Brainstorm Possible Answers

The second step concerns “brainstorming” about possible answers and using “free association.” This is a creative process that includes consulting with experts and primarily involves free association with the aim of generating as many explanations as possible in response to a question. The planners can later disregard explanations that are poorly supported in the literature. In formulating these provisional explanations, applied behavioral scientists typically draw on theoretical and empirical knowledge, whether consciously or not. Doing so is unavoidable at this stage, but the brainstorming should be as open as possible and should not be limited to data- or theory-informed. Only in the next steps, empirical findings (of existing research in step 3 and new research in step 5) and theoretical support (step 4) are incorporated to avoid haphazard decisions based on a brainstorm only. Also, the planning group should then bear in mind that: (1) an explanation should describe a process (an explanation of causation), and (2) an explanation should be plausible. For example, socioeconomic status may be an important contextual factor—or even a root cause—of certain behaviors, but it may need to be explored further in order to better describe a process that explains behavior and thus identify factors that are part of the causal process but are more proximal to the behavior and also more easy to change [e.g., attitudinal beliefs; (15)]. It may be useful to represent the explanation in a process model that shows causation (7).

### Step 3. Review Empirical Findings From Published Research

The next step is to support or refute provisional answers to the questions that the planning group has asked with empirical and theoretical evidence, starting with reviewing findings from published research. The idea behind this is to disregard explanations that are poorly supported in the literature. We suggest to start searching for reviews that have already been conducted. There are many sources available in the burgeoning field of systematic reviews and evidence-based public health that are worthwhile to consult before looking for individual studies. When appraising available reviews, or conducting a new one [see (15) for basic how-to guidance], it is warranted to at least understand the nature of the numerator (what studies are used in the evidence summary) in terms of the denominator (what studies were conducted or reported), and to be aware of the variation that exists in the quality of evidence. Of course, the latter also applies when assessing individual studies. We would like to reiterate that Core Processes should be followed in this order. For example, it is unwise to use general theories aimed at explaining behavior if there is ample evidence available on determinants regarding the specific behavior of interest.

### Step 4. Find Theoretical Support Using the Topic, Concepts, and General Theories Approaches

The search of the literature is focused, for example, on a specific behavior, or target group or culture. However, it might be that there is limited literature available (e.g., regarding a certain behavior or target group) or that the literature is limited in scope (e.g., focusing on a limited number of explanations). The next step, therefore, is to find theoretical support for the provisional explanations and to make the provisional list of answers as extensive as possible before conducting new research (i.e., step 5) and making decisions (i.e., step 6).

Theories can be defined as formal and abstract statements about a selected aspect of reality (16). As a consequence of their very nature, theories are always a reduction of reality. This is not a shortcoming, but rather a definition, which is important to keep in mind when using theory in addressing problem-driven problems. Real-life problems are—by definition—complex; otherwise, they would already have been solved without the need to involve researchers. It follows, then, that a multi-theory approach is required [(11), p. 25] in order to further understand and solve real-life problems. This is also why intervention studies do not necessarily lead to improvements of a single theory (17). From this perspective, applying theory to real life problems can be likened to completing a jigsaw puzzle with various theories fitting together to provide an explanation or answer to a planning question (18). The argument that one theory—for example, the Reasoned Action Approach—cannot explain all the possible variances in behavior or behavior change is therefore no reason to discard the theory altogether (16). Not being able to explain all variance in behavior could only be held against a “Theory of Everything,” and there are good reasons why such a theory is undesirable (18).

In a problem-driven context, all theories, theoretical models, and concepts are potentially useful within the parameters that the theory describes (7). Moreover, there are common and unique elements regarding each theory (19, 20). There are three approaches to finding theories: the topic, concept and general theories approaches; these should be utilized in combination but also in that order. Limiting the pool of candidate theories too soon may lead to inadequate answers or, worse, it may lead to conclusions being drawn that are counterproductive.

#### The Topic Approach

Going back to the literature review, the planning group needs to look specifically for theoretical concepts and frameworks that have been used to design the reported empirical studies and/or explain the findings. They then assess these theories in terms of how useful they are for providing additional answers to the formulated question.

#### The Concept Approach

A second approach to find theory-informed answers to the question being asked is to examine concepts that are generated during brainstorming sessions in the second step. It is likely that the ideas resulting from these brainstorming sessions are initially stated in lay terms, but there may be advantages to relabeling them with their theoretical labels. The information that can be garnered about a theoretical construct can be more precise than that related to a simple lay concept (e.g., lack of confidence could also be labeled as the theoretical construct self-efficacy). One person cannot be familiar with all potentially useful theories. This is why it is advisable to include individuals from various disciplines in the planning group and it stresses once again that expertise in behavior change (e.g., an applied psychologist) is always required. It is also worth noting that reading comprehensive overviews of theories may aid this process [(11), Chapters 2 and 3; (21–24)].

#### The General Theories Approach

After the topic and concept approaches, a general theories approach involves exploring a theory that may offer additional insight into the question at hand. At this stage, it may be fruitful to consider alternative frameworks that have not been accessed through the other two approaches but that could provide valuable information for further extending and refining the list of explanations. For example, dual process models of human behavior that differentiate between impulsive or automatic decision making and more reasoned routes of planning [e.g., (25)], or theories of self-regulation and self-management [e.g., (26)] may be informative. Referring back to the earlier statement about the strict order in the three approaches to find theories, the general theories approach should be seen as a last resort to prevent falling back in a theory-driven rather than problem-driven approach in tackling societal problems. When there is tension between generalizability and utility of theories, utility should be given preference given the applied nature of the problem-driven approach (27).

### Step 5. Identify and Address the Need for New Research

It is important that the planning group completes the previously described steps instead of jumping straight into research. A very practical reason is that conducting new research requires a lot of resources (in terms of time, expertise, and money). More important, all evidence and insights that are available should be used before conducting new research: it should be clear what omissions and knowledge gaps to address in the research. For example, the planning group may want to know whether certain theoretical constructs that look promising are actually explanatory in relation to their population of interest.

### Step 6. Complete and Assess the List of Possible Answers

At this point, the planning group is ready to summarize and complete the provisional list of answers into a working list of items for which the theoretical and empirical evidence is evaluated as sufficient. The planners will consider the criteria relevance and changeability of the evidence- and theory-based answers.

## Example: Applying Core Processes

The following example nicely illustrates the use of the Core Processes [(11), p. 21–8]. In this example, a group of students in a health education class designed a project to prevent the transmission of HIV and other sexually transmitted infections (STIs) and pregnancy among urban adolescents.

### Step 1. Pose Questions

Over the course of the project, they asked a number of questions, including: (1) *Health problem*. What are the health problems associated with HIV, STIs, and pregnancy in adolescents (ages 13–18) in the USA? (2) *Behaviors*. What are important risk behaviors for the transmission of HIV and STIs, and for pregnancy among adolescents? How do these risk behaviors vary, for example, between boys and girls? (3) *Determinants*. About the *risk* behavior: Why don't adolescent males use condoms when having sex with steady girlfriends? Why do girls have sex with boys who do not use condoms? About the *health-promoting* behavior: Why would girls carry condoms? Why would adolescents discuss condom use with their partners? (4) *Change methods*. What change methods relate to what determinants? How can change methods be translated into appropriate practical applications? (5) *Implementation*. How could such an intervention be implemented?

### Step 2. Brainstorm Possible Answers

Using “free association,” planning group members generate as many explanations as possible that can later be dropped when poorly supported (8). Trained behavioral scientists already know a lot about determinants of behavior and barriers for change and this knowledge should be used. In Table 1, the first column represents the outcome of the brainstorm regarding determinants of condom use.

**Table 1:** List of answers regarding condom use among adolescents [(11), p. 21–8].

**Step 1: Pose questions**			
**Step 2: Provisional list resulting from brainstorming**	**Step 3: Additions from empirical literature**	**Step 4: Theoretical additions**	**Step 5: Additions from new research**
Lack of knowledge about HIV transmission Lack of knowledge about STIs Peers don't use condoms Perception that condoms don't work Attitudes toward condom use Experience with condom use; don't like condoms Gender; males do not want to use condoms Lack of salience—not knowing someone with AIDS Lack of confidence in using condoms	Do not perceive condoms as means of pregnancy prevention Perceive condoms as embarrassing Did not express personal responsibility for having condoms Lower family connectedness Parents' permissive attitudes toward sex Community perceptions of gender inequality in sex Closed communication style Neighborhood characteristics, such as high unemployment Lack of access to family planning services Lack of parental supervision Parental trust	Intention to use condoms Subjective norms Perceived norms Self-efficacy for negotiating and discussing condom use with partner Skills Outcome expectations	Lack of knowledge about HIV or STIs disconfirmed Argument that condoms don't work is an excuse, not a belief Experience with condoms associated with embarrassment Teens wanted to be more skillful Girls and boys both expressed that condoms were the responsibility of the other gender Perception of no risk of HIV with only one partner (mistook “serial monogamy” for monogamy)
**Step 6: Complete and assess the list of possible answers**			

### Step 3. Review Empirical Findings From Published Research

The second column in Table 1 presents the outcomes of the review on the evidence supporting the results of the brainstorm. The intervention planners identified empirical evidence for some issues related to unprotected sex that were not already brainstormed, for example not perceiving condoms as a means of pregnancy prevention (28) or perceiving condoms as embarrassing (29, 30). The planning group also identified a number of studies that reported the relationship between unsafe sex and various theoretical constructs (listed in the third column): intention to use condoms and perceived norms (28, 31) and self-efficacy in terms of negotiating and discussing condom use with partners (29, 32). Ideally, those concepts (as depicted in Table 1) should be specified at the level of beliefs, for example the specific beliefs that underlie an attitude or self-efficacy (33). The planning group also became interested in information on the wider social context. For example, community characteristics—such as a high proportion of families living below the poverty line, a low level of education, and high unemployment—were found to be strongly related to teenage pregnancies (34).

### Step 4. Find Theoretical Support Using the Topic, Concepts, and General Theories Approaches

#### Topic Approach

The literature review identified a meta-analysis study on the psycho-social determinants of condom use in heterosexual populations by Sheeran et al. (35). In the introduction and discussion sections, these authors refer to different psychosocial theories of (health) behavior such as the Health Belief Model (36), the Theory of Planned Behavior (37), and the Aids Risk Reduction Model (38). By studying these theories in detail, additional answers can be added to the list of potential explanations that are supported by theories of human behavior (Table 1, third column).

#### Concept Approach

Lack of confidence appeared on the original list. This concept could also be labeled as the theoretical construct self-efficacy. By further exploring the construct of self-efficacy in the literature (39, 40), the planning group may then also discover that self-efficacy is closely related to skills, perceived norms, and outcome expectations. As a result, they could add perceived norms and skills for negotiating condom use and applying a condom to the list (Table 1, third column). In this additional exploration of the theoretical literature, the group may encounter methods for influencing self-efficacy and think ahead in terms of how to apply this in the intervention. None of this useful information would have been available if the group had not related confidence to the concept of self-efficacy and studied the underlying theoretical framework.

#### General Theories Approach

The planning group could have used the general theories approach to access Social Cognitive Theory (41), but of course the topic and concept approaches would most likely also have led the planning group to this theory.

### Step 5. Identify and Address the Need for New Research

In the next step, the planning group needed more information from their priority population about the items on the provisional list in order to determine whether these proposed factors were relevant to their particular population. To this end, the group conducted focus groups with seventh- and eighth-grade students from the priority population. The new data called into question the notion of a lack of knowledge about HIV or STIs in the adolescent population. Interestingly, the adolescents also felt that the argument “condoms don't work” is more of an excuse and less of a belief about their effectiveness. The adolescents who had tried condoms expressed some embarrassment with the process of using condoms and a need for a greater level of skills and self-efficacy. With this new information (Table 1, fourth column), the planning group was able to proceed to the final step.

### Step 6. Complete and Assess the List of Possible Answers

**Figure 2:**
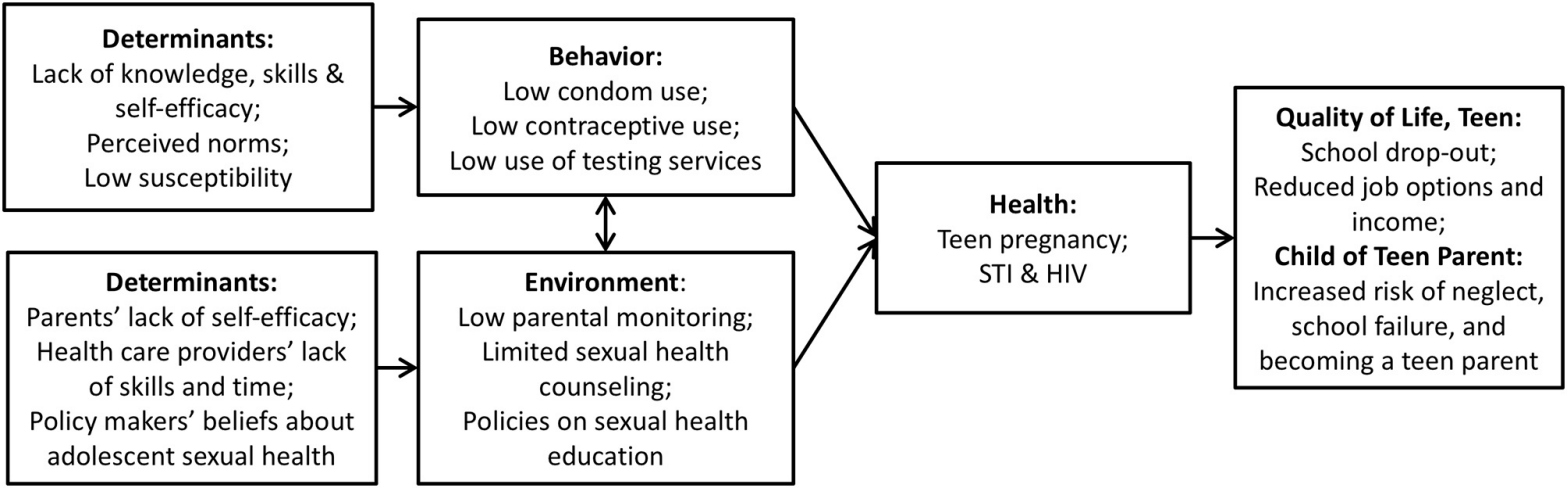
Logic Model with Relevant and Changeable Determinants [adapted from (11), p. 259].

In the final step, the planning group completes the provisional list of answers and summarizes it into a working list for which the evidence is sufficient. The provisional list of answers from the brainstorm is thus followed up by a list of answers for which theoretical and empirical support has been sought. In step 6, the planning group then decides whether the evidence is sufficient by assessing the answers in terms of relevance *and* changeability. *Relevance* refers to the strength of the evidence for the association between the determinant and the behavior. Crutzen et al. (42) provide a practical approach to select determinants based on visualization of confidence intervals for the means and correlation coefficients for all determinants simultaneously. *Changeability* refers to the strength of the evidence suggesting that the proposed change can be realized by an intervention. Whenever possible, judgments regarding changeability should be based on evidence from the research literature (43). However, when data regarding changeability are scarce, such judgments have to rely on a theoretical or conceptual basis. Behavior change expertise is then needed to make judgments regarding changeability. See Figure 2 for a logic model concerning the overarching project from which this example was derived.

## Core Processes for Selecting Change Methods

For brevity and consistency reasons, the example used above to illustrate the Core Processes in answering questions with empirical and theoretical support mainly concern selection of determinants (i.e., addressing “why” questions). We would like to stress that Core Processes also need to be used to select change methods for behavior change or to systematically plan implementation and evaluation of interventions (11). In other words, to also address “how” questions. The focus of the questions then shifts to potential solutions or theory- and evidence-based change methods, for example: How can we encourage specific subgroups of adolescents to use condoms? How can change methods be translated into appropriate practical applications? In relation to a solutions or methods question, answers that remain on the list after engaging in all Core Processes will be methods that have been shown to produce significant change in similar situations. Kok et al. (44), for example, provides tables with theoretical methods (and their limiting conditions) for every major determinant and for all higher environmental levels, i.e., interpersonal, organizational, community, and policy levels. It is important to bear in mind that theory-based methods are only effective under certain limiting conditions, i.e., the parameters for effectiveness (12). When these parameters are ignored—or lost in translation from behavior change method to practical application—effective behavior change is undermined and the intervention may even result in unintended or counterproductive effects (45). Parameters for effectiveness are another example stressing that although the required expertise within multidisciplinary planning groups may vary based on the problem that is addressed, expertise in behavior change (e.g., an applied psychologist) is always required.

## Conclusion

Applied psychology is a scientific discipline in which different kinds of societal problems and issues are addressed. The garnering of expert knowledge, the search for existing literature, the selection and correct application of appropriate theories, and the collection of additional research data are essential for the systematic development of any intervention. It is, however, often unclear exactly how and when these processes should be used in problem analysis and solving. Core Processes are presented as a helpful and systematic way to answer questions raised in different phases/steps of planning frameworks. So, Core Processes are not a planning framework on their own, but a way to address questions relevant to problem definition and solution using evidence, theories, and research.

## Author's Note

We have presented this work at the 2019 conference of the European Health Psychology Society and an abstract similar to this paper was included in the proceedings of that conference (46).

## Author Contributions

All authors listed have made a substantial, direct and intellectual contribution to the work, and approved it for publication.

## Conflict of Interest

The authors declare that the research was conducted in the absence of any commercial or financial relationships that could be construed as a potential conflict of interest.
